# Primary lung cancer with delayed drug-induced interstitial lung disease after neoadjuvant chemoimmunotherapy: a case report

**DOI:** 10.1186/s44215-026-00247-4

**Published:** 2026-02-18

**Authors:** Hideomi Ichinokawa, Takashi Sowa, Takashi Yaguchi, Yukio Watanabe, Kazuya Takamochi, Kenji Suzuki

**Affiliations:** 1 Department of General Thoracic Surgery, Juntendo University Shizuoka Hospital, 1129, Nagaoka, Izunokuni, Shizuoka 410-2295 Japan; 2 Department of General Thoracic Surgery, Juntendo University Hospital, Tokyo, Japan

**Keywords:** Lung cancer, Neoadjuvant chemoimmunotherapy, Immune-related adverse events, Surgery, Interstitial lung disease

## Abstract

**Background:**

Neoadjuvant chemoimmunotherapy is becoming a mainstream treatment for primary lung cancer; however, delayed immune-related adverse events occurring at 3 months or more after discontinuation of treatment have not been reported in this patient population.

**Case presentation:**

A 64-year-old man underwent bronchoscopic biopsy, revealing a lung adenocarcinoma (cT4N0M0, stage IIIA), and was treated with three courses of carboplatin, pemetrexed, and nivolumab as neoadjuvant chemoimmunotherapy. Four months after the first course of treatment, the patient underwent a left upper lobectomy with lymph node dissection. Although postoperative hoarseness occurred as a complication, he was discharged on postoperative day 12. On postoperative day 56, the patient developed shortness of breath. An increased inflammatory response and infiltrates in the right lower lobe suggested aspiration pneumonia; however, his condition failed to improve after 1 week of antibiotic therapy. Elevated KL-6 levels and worsening of a right lower lobe interstitial shadow were observed. On day 104 after immunotherapy, the patient was diagnosed with delayed drug-induced interstitial lung disease, and steroid therapy resulted in improvement.

**Conclusions:**

In patients with a history of immune checkpoint inhibitor (ICI) administration, attention must be paid to the occurrence of immune-related adverse events, regardless of the duration of ICI administration or the time elapsed since discontinuation, and when diagnosed, treatment should be initiated immediately in accordance with guidelines.

## Background

Similar to advanced and locally advanced cancers, chemoimmunotherapy is now commonly being used for perioperative treatment of early-stage lung cancer. In several phase III trials, including the CheckMate-816 trial [1], KEYNOTE-671 trial [2], and AEGEAN trial [3], preoperative treatment with immune checkpoint inhibitors (ICIs) in patients with operable clinical stage IIA–IIIB non-small cell lung cancers (NSCLC) resulted in significant improvements in event-free survival and pathological complete response rates, both tumor endpoints, compared with chemotherapy alone. Although delayed autoimmune events occurring months to years after discontinuation of immunotherapy have been described in other types of cancer [4, 5], no cases of late-onset interstitial lung disease (ILD) following preoperative chemoimmunotherapy for primary lung cancer have been reported.

Herein, we report a case of a delayed exacerbation of immune-mediated ILD that occurred more than 3 months after the final dose of neoadjuvant chemoimmunotherapy.

## Case presentation

**Fig. 1:**
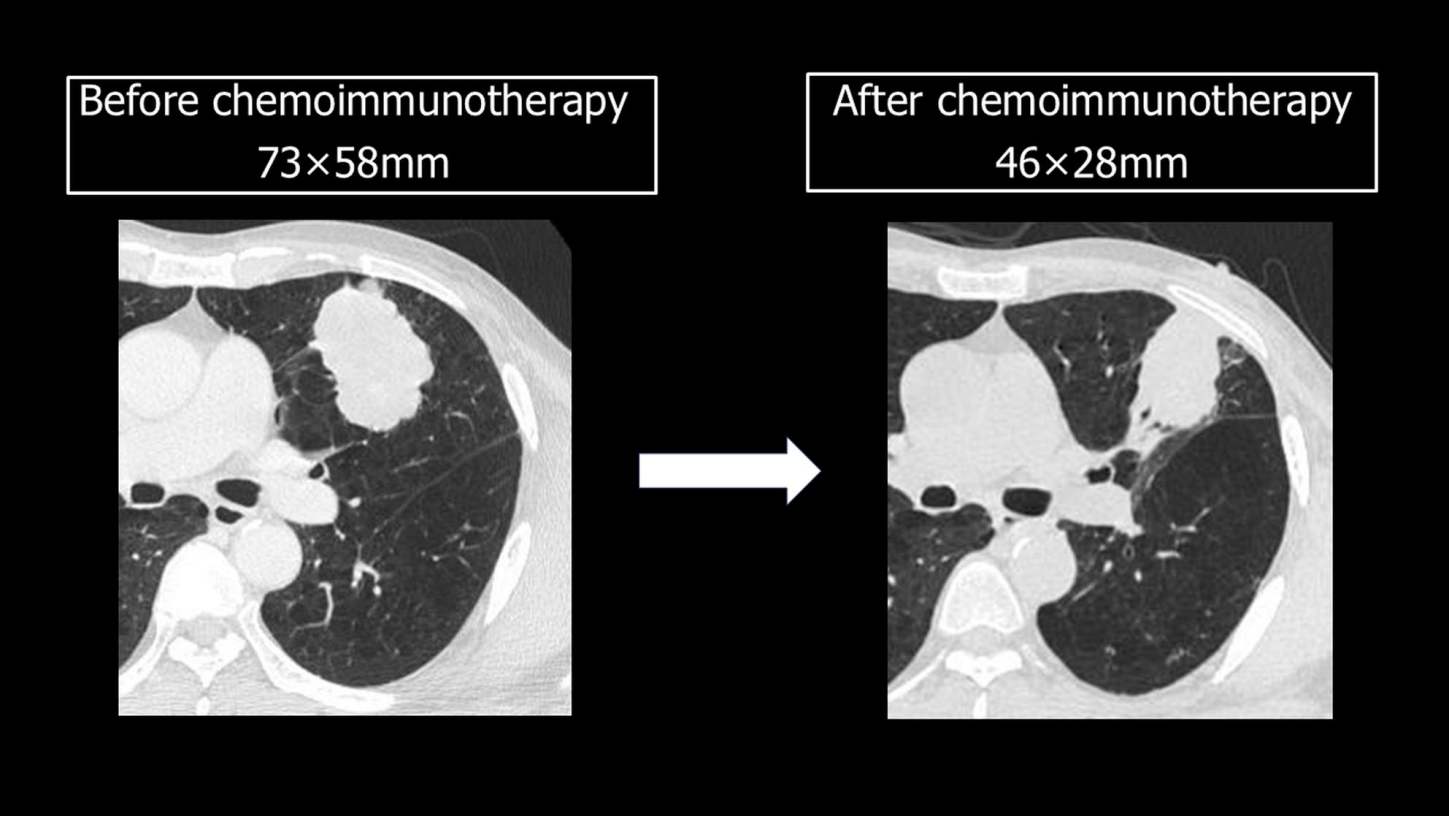
Tumor size before and after neoadjuvant chemotherapy. Chest computed tomography imaging showing the size of the tumor before and after neoadjuvant chemoimmunotherapy and immunotherapy. A 73 × 58-mm mass was observed in the left upper lobe, which shrank to 46 × 28 mm after chemotherapy

A 64-year-old man was referred to our hospital’s Department of Respiratory Medicine in July 2024 following the identification of a tumor in his left upper lobe. In August 2024, lung adenocarcinoma was diagnosed by a transbronchial lung biopsy (clinical stage T4 [tumor diameter = 73 mm] N0M0, stage IIIA). As preoperative chemoimmunotherapy, the patient underwent three courses of carboplatin, *pemetrexed*, and nivolumab starting in September 2024, resulting in a partial response where the tumor size decreased from 73 × 58 mm to 46 × 28 mm (Fig. 1).

We performed a left upper lobectomy and lymph node dissection (ND 2a-2) in December 2024. The operative time was 311 min, with a blood loss of 115 mL. The postoperative pathological diagnosis was a *large-cell neuroendocrine carcinoma with adenocarcinoma components* (y-pathological stage T2b [tumor diameter = 50 mm] N0M0, stage IIB; treatment response rate: 30%). Postoperative complications included hoarseness, caused by a recurrent laryngeal nerve palsy, and hypoxemia, requiring the initiation of home oxygen therapy. The patient was discharged on postoperative day (POD) 12.

**Fig. 2:**
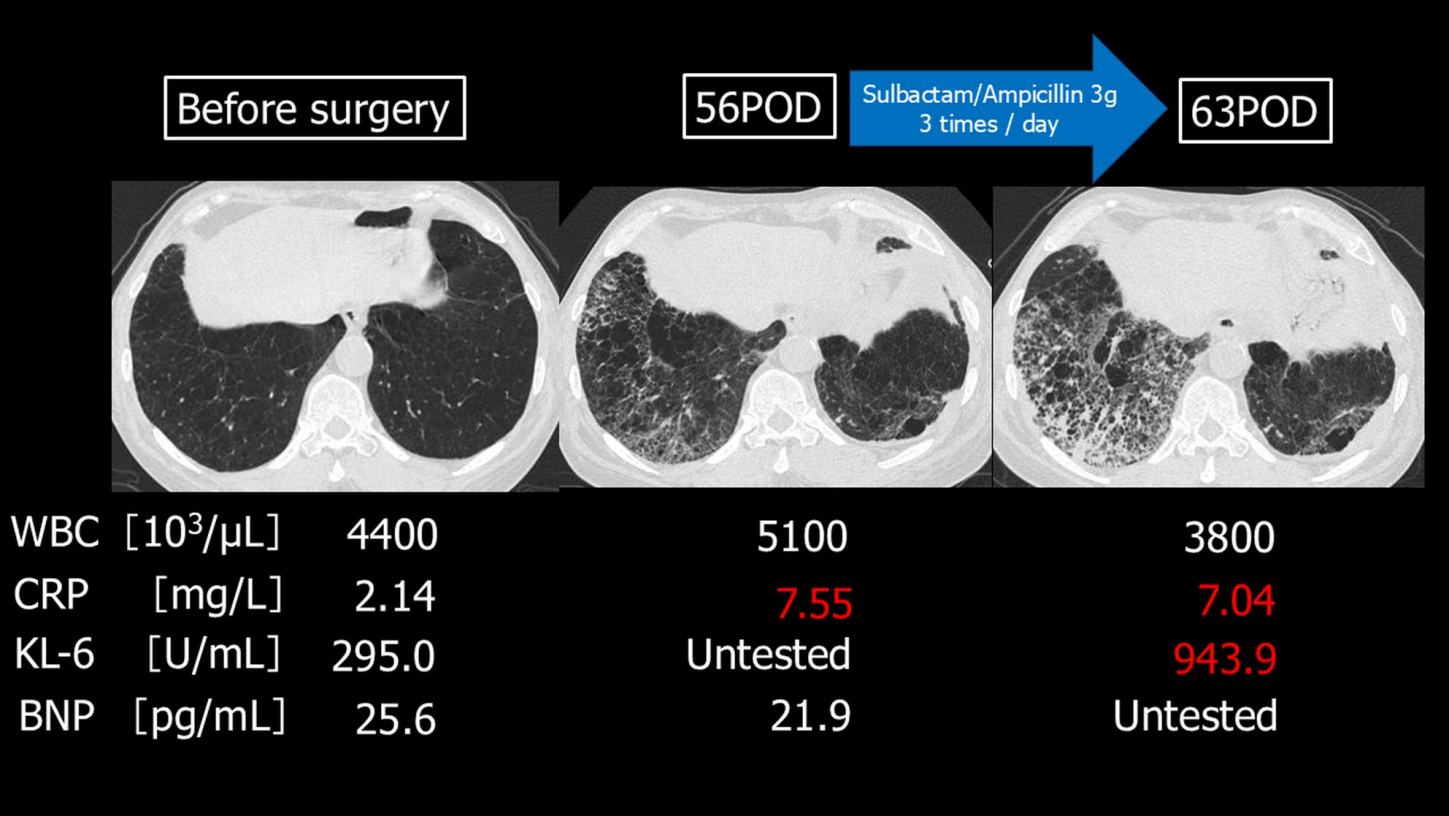
Preoperative and postoperative computed tomography imaging and biochemical test results This image shows the progression of the lower lobe pneumonia on chest computed tomography images before and after surgery, along with the corresponding biochemical test results. On POD 56, pneumonia in the right lower lobe was observed, and the C-reactive protein level was elevated. By POD 63, the pneumonia in the right lower lobe had worsened. POD: Postoperative day

On POD 56, the patient presented to our hospital with shortness of breath. Chest computed tomography (CT) imaging revealed an infiltrate in the right lower lobe, and elevated inflammatory markers (white blood cell count [WBC], 5.1 × 10^3^/µL; C-reactive protein [CRP], 7.55 mg/L) were observed, leading to a diagnosis of aspiration pneumonia (Fig. 2). One week of antibiotic therapy (sulbactam/ampicillin 3 g TID) was administered without symptomatic improvement. At this point, although no increase in his inflammatory response (white blood cell count [WBC], 3.8 × 103/µL; C-reactive protein [CRP], 7.04 mg/L) was observed, KL-6 levels were found to be elevated (943.9 U/mL), and a repeat chest CT scan revealed worsening of the interstitial opacities in the right lower lung field.

**Fig. 3:**
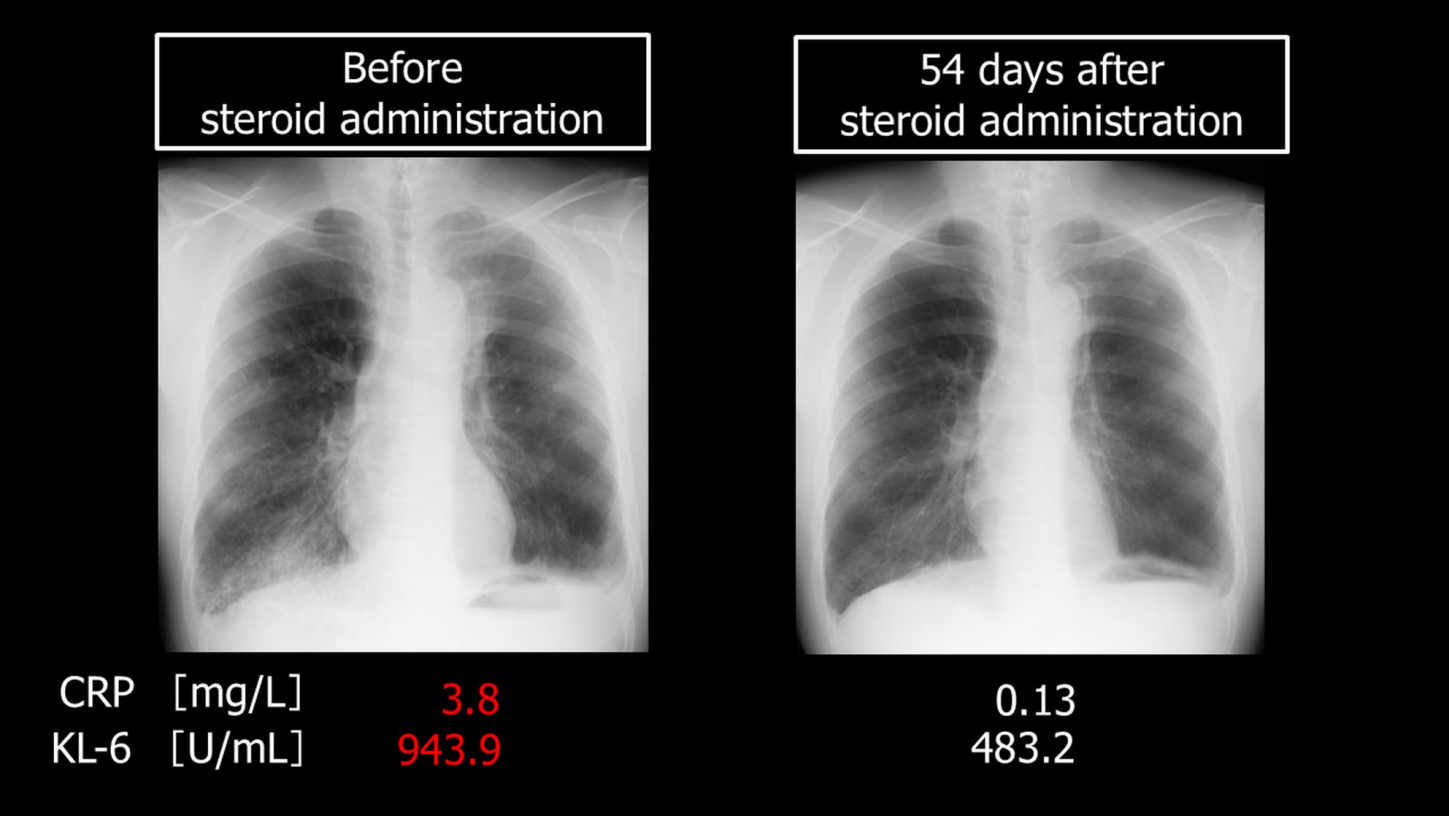
Biochemical test results and chest X-ray imaging before and after steroid administration. This image shows chest X-rays taken before and after steroid administration, along with accompanying biochemical tests. Steroid administration resulted in improvements in areas of decreased permeability in both lower lung fields, as well as an improvement in the inflammatory response and KL-6 levels

**Fig. 4:**
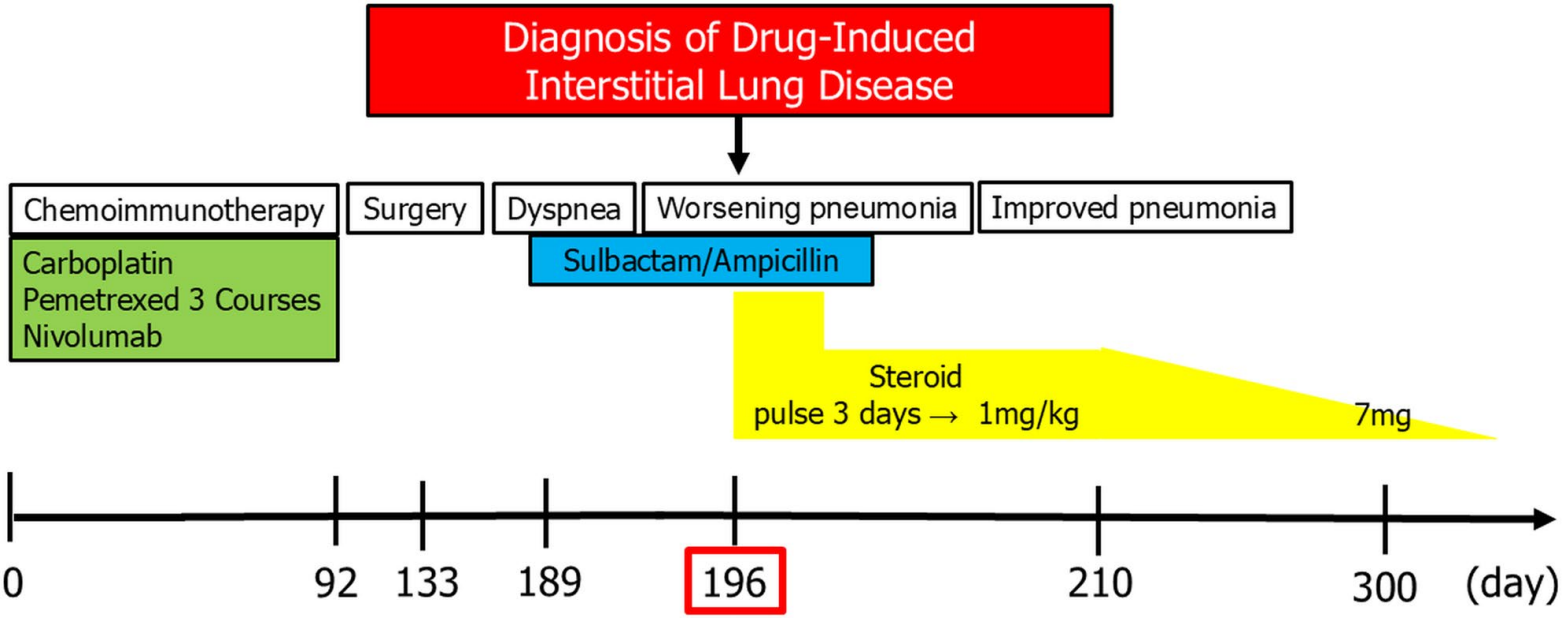
Patient’s clinical course after chemoimmunotherapy administration. This image shows the patient’s clinical course after chemoimmunotherapy. The patient was diagnosed with drug-induced interstitial lung disease at 196 days after the first administration of immune checkpoint inhibitors and 104 days after discontinuation of chemoimmunotherapy

Based on these findings, we suspected late-onset, drug-induced ILD. We continued antibiotic therapy for 1 week and also immediately initiated steroid pulse therapy for 3 days (1000 mg), followed by steroid maintenance therapy (1 mg/kg). His chest X-ray findings and CRP and KL-6 levels gradually improved (Fig. 3). Ten months after surgery, the steroid dose was reduced to 7 mg/day. Hoarseness improved, he no longer required supplemental oxygen, and there have been no recurrences. The patient has been continuously monitored as an outpatient (Fig. 4).

## Discussion and conclusions

ICIs enhance the activity of antitumor T cells by inhibiting various immune checkpoints, including the programmed cell death (PD1) receptor, and have demonstrated clinical efficacy for patients with various types of cancer. However, there are concerns about the risk of immune-related adverse events (IrAEs) when using ICIs, which can lead to complications requiring permanent treatment, such as hormone replacement for endocrine disorders or insulin administration for type 1 diabetes, as well as serious adverse events, including acute exacerbations of ILD and myocarditis, making the recognition and management of adverse events very important.

Couey et al. retrospectively reviewed clinical trials involving immunotherapy and summarized 23 cases in which IrAEs occurred at 90 days or more after treatment discontinuation [6]. The median number of immunotherapy doses administered was four, and the median treatment-free period after discontinuation was 6 months (range: 3–28 months). One hypothesis supporting this idea is that, despite the serum half-life of nivolumab being approximately 12–20 days, the PD-1 receptor occupancy rate of T cells has been shown to remain stable at approximately 80% up to 90 days after a single dose [7]. After three doses of nivolumab, the occupancy rate remains at 40% at more than 8 months after the last dose. As with clinical responses, which are independent of dose and treatment duration, this information suggests that the onset of immunotoxicity also varies depending on the type of treatment.

Furthermore, Durbin et al. reported that, among patients hospitalized for IrAEs after immunotherapy, 14.7% developed the condition within 6–12 months after the first ICI administration, and 10.8% developed the condition at more than 1 year after the first administration [8]. The cumulative probabilities of IrAE development after immunotherapy initiation has been reported to be 42.8%, 51.0%, and 57.3% at 6, 12, and 24 months, respectively [9]. These findings suggest that some patients may need to be hospitalized for IrAE management up to several years after starting ICI therapy.

In this case, we initially suspected an aspiration pneumonia due to the patient’s hoarseness, a postoperative complication, and we administered antibiotics as an initial treatment, even though the postoperative course was delayed. However, 1 week later, chest CT scans showed worsening of the interstitial pneumonia in the right lower lobe. Although the WBC count was not elevated, the increased CRP and KL-6 levels were persistent, leading us to consult a respiratory medicine specialist. Drug-induced interstitial pneumonia was immediately suspected, and treatment was initiated in accordance with the ASCO [10] and ESMO [11] clinical practice guidelines. This highlights the need for ongoing monitoring of potential irAEs by medical professionals, regardless of the time elapsed since ICI administration.

Herein, we report a case of a patient with delayed drug-induced ILD that occurred 6 months after his first dose of preoperative chemoimmunotherapy for primary lung cancer and 3 months after treatment discontinuation. Clinicians should always pay close attention to the development of IrAEs regardless of the duration of ICI administration or the time elapsed since discontinuation.

## Data Availability

All data generated or analyzed during this study are included in this published article.

## Abbreviations

CRP: C-reactive protein
CT: Computed tomography
ICI: Immune checkpoint inhibitors
ILD: Interstitial lung disease
IrAEs: Immune-related adverse events
NSCLC: Non-small cell lung cancer
POD: Postoperative day
WBC: White blood cell
